# Mild hypothermia enhances regenerative gene expression in late-stage neural precursors

**DOI:** 10.3892/mi.2025.252

**Published:** 2025-07-16

**Authors:** Laura Dina Mitrečić, Eliana Eunjoo Park, Aya El-Hajj, Dinko Mitrečić

**Affiliations:** 1 Laboratory for Stem Cells, Department of Regenerative Neuroscience, Croatian Institute for Brain Research, School of Medicine, University of Zagreb, HR-10000 Zagreb, Croatia; 2 School of Medicine, University of Pavia, I-27100, Pavia, Italy

**Keywords:** neural stem cells, hypothermia, Nestin, Map2, neuronal plasticity

## Abstract

The present study investigated the effects of temperature modulation on neural precursors at various developmental stages. A well-proven neural stem cell model was employed and cells were exposed to hypothermia (32, 30 and 28˚C) and hyperthermia (39˚C) for periods of time. While deviations from the physiological temperature (37˚C) affected cell survival and generally reduced cell numbers, a distinct response was observed in late-stage precursors (day 14). Mild hypothermia (32˚C) at this stage preserved cell viability and increased the expression of Nestin, a marker of immature neuronal cells, while decreasing expression of the Map2, a marker of mature neurons. These combined findings suggest a potential dedifferentiation process and an enhanced regenerative capacity. Additionally, the levels of genes associated with cell adhesion, migration and differentiation (Ncam1 and Itgb1) were upregulated by mild hypothermia at day 14. On the whole, these findings highlight the differential response of neural precursors to temperature and suggest that hypothermia timing may be crucial for optimizing therapeutic strategies in neonatal brain injury.

## Introduction

While the majority of human neurons are generated prenatally, substantial neuronal growth, remodeling and differentiation continue during the first 2 years of life (1-3). Postnatal neurogenesis in humans is largely confined to the subventricular zone; however, protracted neuronal migration occurs, notably within the cerebral cortex and cerebellum, which are regions crucial for learning and motor coordination (4). In preterm infants, premature exposure to the extrauterine environment disrupts the typical trajectory of neuronal maturation. This disruption affects all stages of neuronal development, increasing the risk of long-term neurodevelopmental and behavioral disorders. Individual resilience to these risks varies and may be enhanced by early interventions (5,6).

Regardless of gestational age at birth, neonatal hypoxic-ischemic encephalopathy (HIE) represents a severe complication. HIE is a form of brain injury resulting from perinatal oxygen deprivation and reduced cerebral blood flow. It is a leading cause of neonatal brain damage, with an estimated incidence of 2-3 per 1,000 live births in developed countries (7-9). At the molecular level, HIE initiates a cascade of cellular events, including energy failure, glutamate excitotoxicity, calcium overload and oxidative stress, collectively contributing to neuronal injury. Secondary inflammation and apoptosis further exacerbate this damage, highlighting the complex pathophysiology of HIE (10).

Among the various strategies evaluated to mitigate perinatal brain injury, including erythropoietin, stem cell therapies and melatonin, therapeutic hypothermia has exhibited the most consistent clinical efficacy. Cooling the body to 32-34˚C decelerates the metabolic processes and attenuates key pathological mechanisms, such as inflammation, oxidative stress, and apoptosis. Consequently, therapeutic hypothermia is widely considered the standard of care for infants with moderate to severe HIE, significantly reducing mortality (11-13). Furthermore, hypothermia has been shown to improve neurodevelopmental outcomes (14-16).

While previous research has primarily focused on the effects of hypothermia on mature neurons exposed to hypoxia, its effects on neurons at various stages of maturation, including those not directly affected by oxygen deprivation, are less well understood. Given that the neonatal brain, particularly in preterm infants, contains a substantial population of immature neural cells, the response of these cells to both hypothermic and hyperthermic conditions represents a critical area of investigation. The present study investigated the effects of temperature modulation (hypothermia and hyperthermia) on neurons at different stages of maturation. Using a well-established neural stem cell model at various differentiation stages, previously validated in several publications (17-19), the present study examined the differential responses of immature and mature neurons to short-term hypothermia. The findings presented herein provide novel insight into how neural cells at different developmental stages respond to environmental stressors, with implications for optimizing therapeutic strategies for neonatal brain injury.

## Materials and methods

### Isolation and differentiation of neural stem cells

The neural stem cells used in the present study were obtained from the bank of the Laboratory for Stem Cells (School of Medicine, University of Zagreb, Zagreb, Croatia). No additional animals were sacrificed for the purposes of the present study. Isolation procedure, performed in the Laboratory for Stem Cells and described in some of the authors' previous publications (17-19), included 14.5-day-old C57/BL6 albino mouse embryos. They were obtained by mating 3-4-month-old animals. After confirming pregnancy, on day 14, the animals were sacrificed by cervical dislocation. After opening the abdominal cavity and isolating embryos from the uterine wall, embryos were first separated from the amniotic layer and their telencephalic wall was then cut into small sections prior to exposure to the digestive enzymes. Cells were grown in proliferation medium which contained 1% N2 (17502-048, Gibco, Thermo Fisher Scientific, Inc.), 1% Pen/Strep (penicillin/streptomycin, 5,000 U/ml; 15070063, Gibco, Thermo Fisher Scientific, Inc.), 2% B27 (17502, Gibco, Thermo Fisher Scientific, Inc.), 20 ng/ml epidermal growth factor (EGF; PMG8041, Gibco, Thermo Fisher Scientific, Inc.), 10 ng/ml basic fibroblast growth factor (bFGF; PMG0035, Gibco, Thermo Fisher Scientific, Inc.) and 5 mM HEPES (H0887, MilliporeSigma). After a sufficient number of cells was obtained, which was usually in the range of 2-5 million, their differentiation was achieved in the plates covered with 50 µg/ml poly-D-lysin (PDL; P6407, MilliporeSigma) and 10 µg/ml laminin (L2020, MilliporeSigma). Differentiation medium, which induced the differentiation of immature cells into neurons, was comprised of 1% N2, 1% Pen/Strep, 1% FBS (15070063, Gibco, Thermo Fisher Scientific, Inc.), 2% B27+ (A3582801, Gibco, Thermo Fisher Scientific, Inc.) and 5 mM HEPES. The use of these cells was already described and validated in previous publications (17-19).

After obtaining neural stem cells by the enzymatic dissection of the neutrospheres, cells attached on coated coverslips were grown in an incubator up to 14 days. Of note, five different groups of each maturity stage were formed, which corresponded to following temperatures: 32, 30 and 28˚C (three groups of hypothermia), 39˚C (hyperthermia, and the control group (37˚C). These temperatures were selected on the basis of clinical experience with applied hypothermia protocols (12-16). The temperature of 39˚C was selected as an average hyperthermic value in pathological conditions. After being exposed to the specific temperature for 48 h, the cells were prepared for staining and immunocytochemistry. In this manner, there were three groups of cells with various levels of maturity [day 2, early progenitors (exposed to different temperatures from day 0 to 2); day 7, mid progenitors (exposed to different temperatures from day 5 to 7); and day 14, late progenitors (exposed to different temperatures from day 12 till day 14)].

### Cell counting

After plating the cells at a density of 50,000 cells per well, four wells were used for counting, with three different fields of view counted in each well. Thus, a total of 12 counts were made for each condition tested. Prior to counting, the cells were examined using the LIVE/DEAD Viability/Cytotoxicity kit (L3224, Thermo Fisher Scientific, Inc.), which enabled the recognition of living cells only.

### Immunocytochemistry

To follow up on cell differentiation and analyze the cells using immunocytochemistry, the cells were grown on coverslips and fixed in 4% PFA (Merck KGaA). Fixation at 4˚C lasted for 10 min. Permeabilization of the cell membranes was performed using 0.2% Triton (Merck KGaA) in PBS for 15 min. Subsequently, the cells were washed with PBS. Blocking was performed with 3% goat serum in PBS at room temperature for 2 h. The primary antibodies used were anti-Nestin (1:100; cat. no. MAB353, Merck KGaA) and anti-microtubule-associated protein 2 (Map2; 1:5,000; cat. no. ab5392, Abcam). After the primary antibodies had attached to their antigens, the cells were washed with PBS. The secondary antibodies were then added and the cells were incubated at room temperature with the secondary antibody for 1 h. The secondary antibody used were the following: goat anti-mouse IgG (H+L), Alexa Fluor™ 488 (1:1,000; cat. no. A11001, Thermo Fisher Scientific, Inc.) and goat anti-chicken IgG (H+L), Alexa Fluor™ 546 (1:1,000; cat. no. A11040, Thermo Fisher Scientific, Inc.). For the counterstain procedure, 1:20,000 DAPI (Roche 10236276001, Merck KGaA) at room temperature was used.

### Reverse transcription-quantitative PCR (RT-qPCR)

Total RNA was isolated from cells using the commercial QIAshredder (79656, Qiagen, Inc.) and the RNeasy Mini kit (74104, Qiagen, Inc.). The RNA concentration was quantified using a NanoDrop ND1000 spectrophotometer (Thermo Fisher Scientific, Inc.). The concentration of RNA for all samples was adjusted to 25 ng/µl. The conversion from RNA to cDNA was accomplished utilizing a high-capacity RNA-to-cDNA kit (4387406, Applied Biosystems, Thermo Fisher Scientific, Inc.). qPCR was then performed using TaqMan Gene Expression Assays for three genes of interest: Neural cell adhesion molecule 1 (Ncam1; Mm01149710_m1, Thermo Fisher Scientific, Inc.), integrin beta-1 (Itgb1; Mm01253230_m1, Thermo Fisher Scientific, Inc.) and cadherin-2 (Cdh2; Mm01162497_m1, Thermo Fisher Scientific, Inc.). As housekeeping genes, β-actin (Mm02619580_g1, Thermo Fisher Scientific, Inc.) and hypoxanthine phosphoribosyltransferase 1 (Hprt1; Mm03024075_m1, Thermo Fisher Scientific, Inc.) were used. The relative quantification was performed using the 2^-ΔΔCq^ method (20).

### Statistical analyses

Statistical analyses were conducted in R Studio version 1.4.1717 (Posit PBC). Comparisons between groups were made using one-way ANOVA followed by Tukey's Honest Significant Difference (HSD) post hoc test. A value of P<0.05 was considered to indicate a statistically significant difference.

## Results

### Short-term exposure to hypothermia has more detrimental effects on early- and mid-stage than on late-stage neural precursors

To investigate the effects of neuronal precursor maturation stage and temperature on cell numbers, the cells were exposed to various temperatures for 48 h on days 0, 5 and 12 of maturation. Cell counts were then performed on days 2, 7 and 14. This allowed for the comparison of the effects of temperature exposure at different maturation stages on cell number.

**Figure 1:**
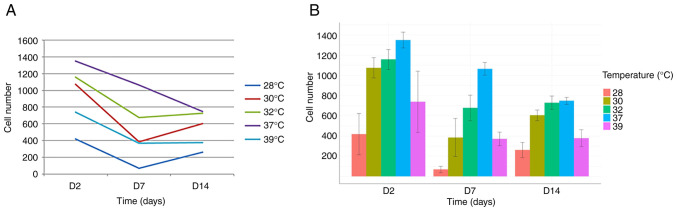
(A) Number of neural precursors measured at the time points of 2, 7 and 14 days, after 48 h of exposure to various temperatures. While exposure to hypothermia and hyperthermia decreased the number of early (D2) and mid-term precursors (D7), compared to the normothermia group, such effects were not so visible in the late precursors (D12-D14), when mild hypothermia (32˚C) was compared to normothermia. (B) Detailed graphs with values and standard deviations for every time point and every temperature. D, day.

**Figure 2:**
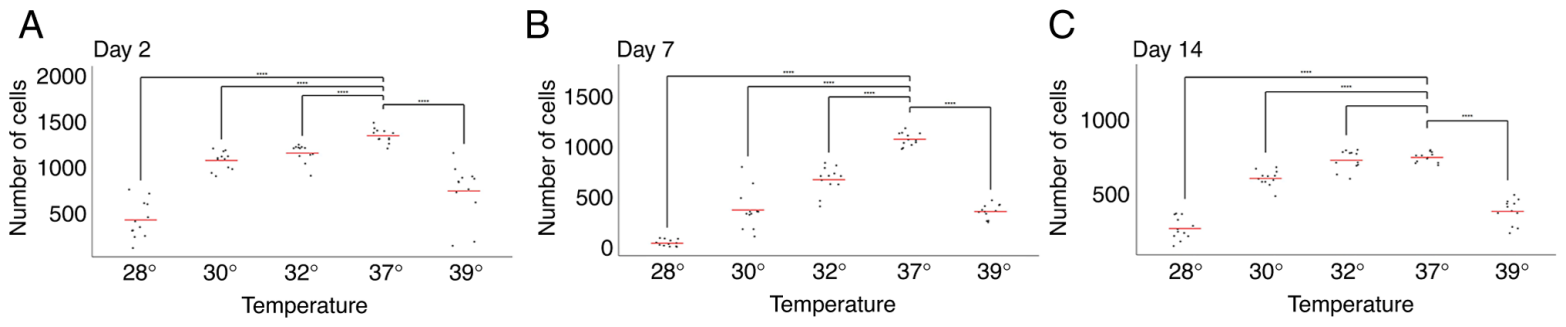
Comparison of single measurements of number of neural precursors for (A) early, (B) mid-term and (C) late-term neural precursors. It is notable that any change in the temperature for culture for 48 h significantly decreased the number of cells, apart from 12-14-day-old precursors exposed to mild hypothermia. ^****^P<0.0001.

The comparison of cell numbers at a physiological temperature (37˚C) revealed a decline in cell numbers between days 2, 7 and 14, consistent with previous observations by our group (17-19) (Figs. 1 and 2).

The analysis of the day 7 time point revealed an accelerated rate of decline in cell numbers following exposure to both hypothermia (at all temperatures tested) and hyperthermia. This suggests that cells exposed to temperature changes between days 5 and 7 responded negatively, resulting in a more pronounced reduction in cell numbers compared to days 2 and 14 (Figs. 1 and 2).

Conversely, and notably, the opposite effect was observed on day 14. Under all three hypothermic conditions (32, 30 and 28˚C), cell numbers on day 14 exceeded those on day 7 (Figs. 1 and 2). This strongly suggests a beneficial effect of hypothermia on cell numbers when applied between days 12 and 14. Furthermore, no significant difference in cell number was observed on day 14 between cells grown at 37˚C and those exposed to 32˚C (Fig. 2).

### The expression of Nestin and Map2, markers of differentiation of neurons, is dependent on the temperature and maturity of cells following exposure to hypothermia

**Figure 3:**
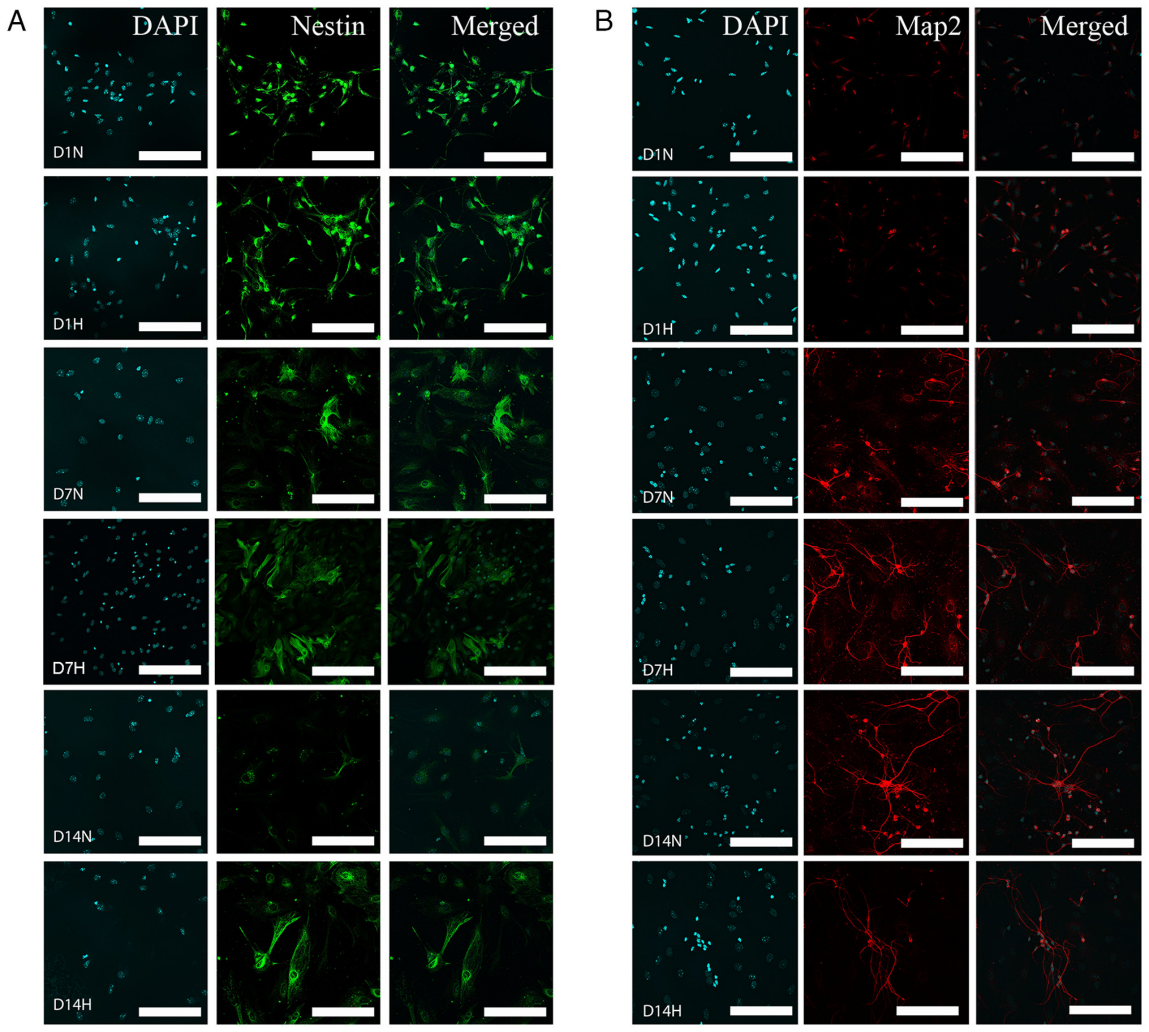
(A and B) Immunohistochemical staining of neural stem cells in three stages of differentiation: Days 1, 7 and 14, exposed to a normal temperature (37˚C; indicated by ‘N’) and to hypothermia (32˚C; indicated by ‘H’). The panels on the left represent DAPI, while those in the middle represent (A) Nestin and (B) Map2. The panels on the right represent the merged image. It is visible that on day 1, Nestin is strongly expressed in both normothermic and hypothermic conditions. On day 7, the Nestin signal is weaker, but hypothermia does not influence it in any notable manner. The most notable finding can be seen on day 14 [compare D14N and D14H in (A)]: While in normal conditions Nestin was almost completely absent, hypothermia re-established the expression of Nestin. The opposite is visible with the expression of Map2, which is reduced in hypothermia on day 14 [compare D14N and D14H in (B)]. Scale bars, 100 µm.

**Figure 4:**
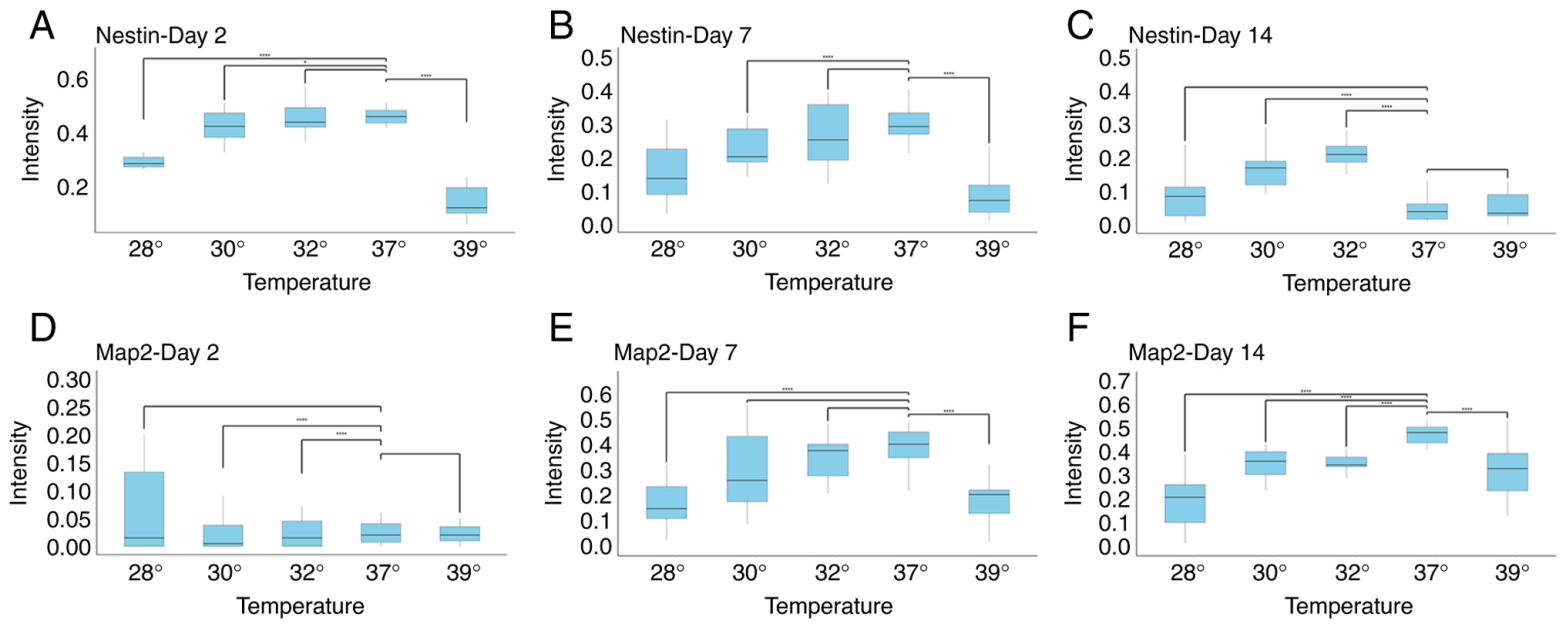
Quantification of immunohistochemical signals of (A-C) Nestin and (D-F) Map2. The most notable finding is shown on day 14: While mild hypothermia increased the expression of Nestin (C), the expression of Map2 was decreased at the same time point (F) ^*^P<0.01 and ^****^P<0.0001. Map2, microtubule-associated protein 2.

To investigate whether changes in temperature influence the expression of markers of neural precursors maturity, the expression levels of Nestin and Map2 were analyzed. As was expected, neural precursors at day 2, and to a lesser extent, at day7, expressed Nestin, while this marker was almost completely absent on day 14 (Figs. 3 and 4). When early precursors were exposed to hypothermia, the expression of Nestin was not markedly altered, apart from the condition of the extreme change of temperature to 28˚C. However, given that the majority of cells at 28˚C were either dead or exhibiting signs of cell death, this finding probably reflects a severe impairment in cell metabolism. Notably, hyperthermia also caused a marked decrease in the expression of Nestin. A similar finding was observed on day 7, where all changes in temperatures decreased the expression of Nestin. On the other hand, the opposite and surprising finding was observed on day 14. While cells at D14 normally no longer expressed Nestin, decreasing the temperature to 32˚C and to a lesser extent, to 30˚C, markedly increased Nestin expression (Figs. 3 and 4).

The analysis of Map2 expression revealed the following: As was expected, Map2 expression was not detected in the earliest stage of neural precursors, and temperature changes did not affect this. At day 7, decreasing the temperature to 32˚C did not influence Map2 expression. A reduction in Map2 levels was observed only at more extreme temperatures, consistent with the findings obtained for Nestin expression. At day 14, both decreases and increases in temperature reduced Map2 expression. Notably, even a decrease to 32˚C reduced Map2 levels, coinciding with a marked increase in Nestin expression at the same differentiation stage (Figs. 3 and 4).

A comparison of all three stages of neuronal maturity revealed that at days 2 and 7, either no significant changes were observed, or a decrease in marker expression was observed, generally being associated with extreme temperature changes, and thus likely representing non-specific effects. At day 14, however, a temperature of 32˚C decreased Map2 levels, but increased Nestin levels, suggesting a form of dedifferentiation (Figs. 3 and 4). This coincides with the observation that cell numbers at day 14 did not significantly differ between 37˚ and 32˚C (Fig. 2).

### Hypothermia increases the expression of genes linked to neuronal plasticity and migration

**Figure 5:**
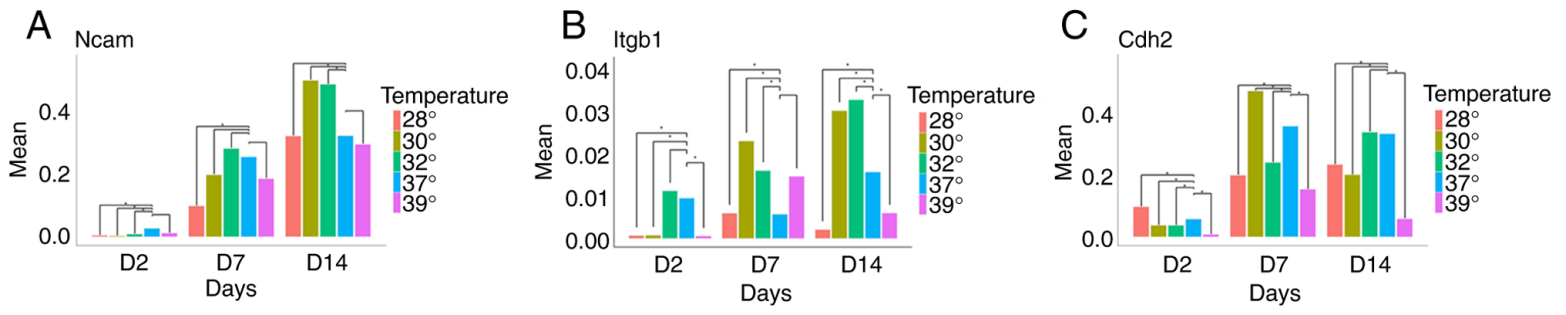
Expression of (A) Ncam1, (B) Itgb1 and (C) Cdh2 at days 2, 7 and 14, in five different temperature conditions. (A) When compared to normothermia, it noteworthy that an increase in Ncam expression was observed following exposure to mild hypothermia on day 14. (B) A similar finding, including on day 7 as well, was found for Itgb1. (C) Cdh2 expression exhibited a different pattern, with an increase in its expression observed only on day 7 when the cells were exposed to 30˚C. ^*^P<0.01. Ncam, Neural cell adhesion molecule 1; Itgb1, integrin beta-1; Cdh2, cadherin-2.

Having observed that temperature changes influence both cell survival and the expression of genes associated with the differentiation stage, the present study then investigated the effects of hypothermia and hyperthermia on the expression of genes related to migration, differentiation, plasticity and regeneration. While Ncam1 was barely detectable in early progenitors, cells at days 7 and 14 strongly expressed this gene. Of note, although temperature changes did not significantly alter Ncam1 expression at day 7, mild hypothermia (32 and 30˚C) significantly increased Ncam1 expression at day 14 (Fig. 5A).

Another gene examined was Itgb1. It was found that temperatures of 32 and 30˚C increased Itgb1 expression at both days 7 and 14, whereas more extreme hypothermia (28˚C) and hyperthermia reduced its expression (Fig. 5B).

The analysis of Cdh2 expression revealed a significant increase only at day 7 when cells were cultured at 30˚C. In all other conditions, a decrease in Cdh2 expression was observed (Fig. 5C).

## Discussion

Therapeutic hypothermia represents a promising intervention for mitigating neonatal brain injury resulting from HIE. By reducing the core body temperature of infants to 32-34˚C for 24 to 72 h, hypothermia decreases metabolic demand and attenuates secondary injury cascades, including inflammation and apoptosis (21). Clinical trials have demonstrated improved survival rates and a reduced risk of severe neurodevelopmental disabilities in term and late preterm infants. However, its efficacy is time-dependent, with maximal benefits observed when initiated within 6 h of birth. Current guidelines recommend hypothermia as the standard of care for moderate to severe HIE in term and near-term infants; however, its use in mild HIE, preterm infants and resource-limited settings remains a subject of ongoing investigation (22). From a molecular and pathophysiological perspective, hypothermia has been shown to inhibit excitotoxicity by reducing glutamate release, thereby preventing excessive calcium influx and subsequent cell death (21). It also downregulates inflammatory pathways, suppressing the release of pro-inflammatory cytokines implicated in cell damage and apoptosis. Regarding apoptosis, or programmed cell death, hypothermia attenuates caspase activation, the enzymatic process responsible for apoptosis (23).

However, the neonatal nervous system, particularly in preterm infants, comprises a heterogeneous population of cells at various stages of maturation. It is therefore plausible that not all cell types are equally susceptible to hypoxic injury. Furthermore, some preterm infants may not experience hypoxia at all, yet hypothermia may be considered a prophylactic intervention to enhance survival prospects. Consequently, the present study investigated the effects of temperature modulation on normoxic cells at different developmental stages. To the best of our knowledge, data regarding the impact of temperature changes on such a broad spectrum of cell types are limited. Therefore, the present study compared the responses of cells at three distinct differentiation stages, unexposed to hypoxic insults, to temperature variations. Notably, deviations from the physiological temperature of 37˚C generally reduced cell numbers. However, the experimental design used herein yielded a key observation: While early- and mid-stage neural precursors exhibited negative responses to both hypothermia and hyperthermia, a distinct benefit was observed at day 14. Under all hypothermic conditions tested, the number of viable cells at day 14 exceeded that at day 7. Furthermore, no significant difference in viable cell numbers was detected between cells cultured at 37 and 32˚C at day 14. This indicates that only at day 14, and only with mild hypothermia (32˚C), were detrimental effects on neural precursors absent. This finding was further investigated by examining the expression of Nestin, a marker of immature cells, and Map2, a marker of mature or nearly mature neurons. Again, late-stage neural precursors displayed a unique response: Mild hypothermia reduced Map2 expression, while simultaneously increasing Nestin expression. This increase in Nestin expression was the sole increase observed in this aspect of the study. Nestin, an intermediate filament protein, is predominantly expressed in stem and progenitor cells during development, particularly within the central nervous system, muscle and other tissues. It is a widely accepted marker for neural stem cells (NSCs) and other multipotent stem cells (24). An elevated expression of Nestin is also observed in reactive astrocytes and other glial cells following central nervous system injury, such as stroke or traumatic brain injury. Notably, Nestin-expressing neurons have been identified in both rats and humans, suggesting that even mature neurons can, under certain conditions, express this protein (25). Moreover, there is evidence to indicate that increasing the expression of Nestin in both neurons and astrocytes contributes to the improved survival of neural tissue affected by pathological condition (26).

Map2 is a protein localized to dendrites and interacts with microtubules, the primary structural filaments of neurons. An increased expression of Map2 in neural precursors is a key indicator of terminal differentiation. Consequently, it has been demonstrated that Map2 inhibition is associated with a reduced rate of differentiation and the maintenance of mitotic capacity (27). The expression of Map2 is known to be modulated by various external factors (28). Moreover, decreased expression of Map2 has been reported in certain pathological conditions (29). The dichotomy regarding the effects of hypothermia on Map2 expression centers on whether a reduced expression of Map2 signals neuroprotection or impaired neuronal maturation. On the one hand, hypothermia has been shown to attenuate the injury-induced loss of Map2, suggesting the preservation of late-stage neural precursors and contributing to neuroprotection after trauma (30). On the other hand, Map2 is critical for dendritic outgrowth, microtubule stabilization and synaptic plasticity; thus, its loss or reduced expression can reflect impaired maturation and pathological dendritic morphology, as observed in various neurodegenerative and neuropsychiatric disorders (29). Thus, while the decreased expression of Map2 under hypothermia may indicate preserved neuronal integrity, a decrease in Map2 expression more broadly could also signify disrupted neuronal development and function. This dual interpretation highlights the complexity of Map2 as both a marker and mediator of neuronal health, where context and timing of expression changes are key to understanding its role.

Given the findings of the present study suggesting that mild hypothermia may postpone the terminal differentiation of neurons and enhance their regenerative potential, the present study investigated the expression of three genes implicated in these processes. Ncam1, widely expressed in neural tissue, is involved in neural stem cell adhesion, migration and differentiation. It plays a crucial role in neurogenesis and synaptic plasticity (31). Generally, Ncam1 upregulation promotes cell adhesion, neural plasticity and migration (32-34). The finding of the present study that mild hypothermia increases Ncam1 expression at day 14 is consistent with the observed increase in Nestin expression and a decrease in Map2 expression, as these changes collectively suggest reduced terminal differentiation and an enhanced capacity for regenerative processes. Itgb1 is a molecule involved in guiding neurons to their appropriate locations during brain development. Furthermore, it participates in neuronal adhesion and migration (35-37). Very similar to Ncam1, the finding of the present study that mild hypothermia increases Itgb1 expression at days 7 and 14 is consistent with the observed increase in Nestin expression and a decrease in Map2 expression. All these changes collectively suggest reduced terminal differentiation and an enhanced capacity for regenerative processes. Cdh2 is a cell adhesion molecule highly expressed in neural tissues. It plays a crucial role in maintaining the neural stem cell niche and influencing NSC differentiation, migration, and synapse formation (38). Unlike Ncam1 and Itgb1, hypothermia did not exert a consistent effect on Cdh2 expression; some temperatures increased and others decreased its expression. Clearly, additional mechanisms are involved in this molecular pathway, and further investigations are required to elucidate these complex interactions.

Regardless of that, it was clearly demonstrated that Ncam1 and Itgb1 play crucial roles in neural stem cell migration and neurite extension. When upregulated, these molecules enhance cellular mobility and neurite formation, while their downregulation produces opposite effects. The mechanistic elements of these phenomena are well-described (39). In addition, it has been reported that hypothermia leads to an enhanced neurite extension in mouse brain slice cultures, which is mediated by a surge in TNF-α levels (40). Moreover, a previous *in vivo* study on rat spinal cord injury found that hypothermia plus NSC transplantation tended to promote graft migration: The combined therapy may promote migration of the transplanted cells at the injury site (41).

While it is known that the use of mice as an animal model and mouse cells are still often used in neuroscience, certain possible limitations of the present study should be mentioned. Mouse NSCs are widely used as models of human neural tissue due to broad genetic and cellular similarities; however, critical species-specific differences can limit their translational relevance (42). Moreover, comparative transcriptomic research has found that a number of orthologous genes exhibit divergent expression or splicing patterns in mouse vs. human neural cells (43). Developmental timelines also markedly differ: Human neurodevelopment is far more prolonged and complex than that in mice; thus, equivalent developmental stages do not align. In other words, the results of the present study are very useful; however, for further progress, more advanced, human brain organoid models, for example, such as the ones previously reported (44) should be used.

## Data Availability

The data generated in the present study may be requested from the corresponding author.
